# Professional Responsibility

**Published:** 1903-09

**Authors:** A. E. Baldwin

**Affiliations:** Chicago, Ill.


### PROFESSIONAL RESPONSIBILITY.[1]

BY A. E. BALDWIN, M.D., D.D.S., LL.B., CHICAGO, ILL.

Mr. Chairman and Gentlemen,—In coming before you on this occasion with this subject for a basis of a paper, I perhaps should premise it by stating that, while this is a hackneyed subject, it is one on which we ought to devote a great deal of thought. We stand before the public and the general profession as the guardians of the entrance to the alimentary canal, and these responsibilities laid upon us determine many things in connection with the general health of our patrons.

These responsibilities begin in our advising our patrons as to the care and carefulness with which the temporary teeth of the

---

[1] Abstract of a paper read before the American Medical Association, Section on Stomatology, New Orleans, May 5 to 8, 1903.

little ones should be protected, as well as advising as to the care of the permanent teeth which obtain in later years.

In a former paper, or rather papers, read before this and other gatherings, the writer has urged upon the general profession, as well as our special department, the care which should be bestowed upon the deciduous teeth. It doubtless is overlooked by many of the general practitioners, and, judging from the writer's observation, by even many in our special field, that the teeth of the growing child should be kept more carefully even than the teeth of the adult. A reason apparent to all is that while in adult life nutrition only to make up for the waste of tissues is necessary, in the growing child there are many reasons why added nutrition should be obtained, chief among which may be mentioned that the waste tissues which must be made up are fully as large in proportion as in the adult, while with the child there is a rapid development of the general system, requiring much additional nutriment. Indirectly, there are many other reasons, among which are these: A child has a vigorous and almost unlimited appetite; if these deciduous molar teeth are allowed to become sensitive, painful, or lost, the mastication of the child is necessarily hampered, and it is an axiom that a child will never masticate food if by so doing pain is caused. They very early in life learn that despicable habit of "bolting" their food with but little or no mastication, if mastication is in any way disagreeable, thus fixing upon them a habit for life which is conducive to many of the ills of indigestion and malnutrition. A still further reason is the fact that all teeth in the mouth, at all ages, have a tendency to move towards the median line of the mouth; hence if one of these deciduous teeth is extracted before its successor is ready to erupt, the teeth behind will move towards the front, thus preventing the proper eruption and proper development and expansion of the arch, and causing the coming in of the teeth in abnormal positions, and also creating irregularity and its subsequent ills.

The writer has always urged upon the general profession and our special department that exceeding care should be given to the deciduous molar teeth, and advising our patrons to urge upon the child a thorough use of the brush, and frequent inspections of these teeth, and that at the least suspicion of decay they should be given immediate professional attention, thus preserving them in a healthful condition, so that mastication can be performed fully and that the ills resulting from "bolting" of food may not invade the stomach of the little patient, bringing upon it all the ills of indigestion, characterized frequently by the recognition of a very nervous little one whose only trouble, probably, is dyspepsia caused by lack of the above attentions. The writer would urge upon our specialty that most careful attention be given to these teeth until the time for them to be replaced by the permanent bicuspids, as well as the very great care that should be observed in attention to the first permanent molar, which is erupted at about the sixth year of age, and the molars which are subsequently erupted posteriorly thereto.

This first permanent molar spoken of we all recognize as the abutment of the arch, and from its location, position, size, and attachments, is the most important tooth of the mouth to be preserved at this or any subsequent period. The loss of this tooth, or lack of attention thereto, as well as the lack of care of the deciduous molars, in the writer's belief produces most of the cases of irregularities and deformities of the lower part of the face, nature having evidently intended that the lower part of the face should be developed and expansion thereto attended by the gradual wedging in and pushing apart of the alveolar process by the eruption of the larger teeth of the permanent set.

The writer would also condemn the custom illustrated by the following experience which lately came to him. A professional gentleman of a neighboring city, whose record for general professional standing is high, had a patient who had been under his care for many years. The family had lately removed to our city, and this patient fell under the writer's professional care. Upon calling his attention to many small cavities in the teeth, he explained that his former attendant had said that he knew there were many small cavities, but that it would be better to wait and fill them later. This, to the writer's mind, is one of the greatest of fallacies, and one which obtains more largely than is generally admitted. The writer's belief is that whenever we have an opportunity to examine a patient's mouth, we should give it a most rigid, careful, and thorough inspection, calling the patient's attention to all of the defects which can be found by such careful inspection, and urging upon them the importance of early attention thereto. Especially would he suggest that if any teeth are left unattended, they should be those which have large cavities therein; in other words, he would urge that if any teeth are given attention by the patient, the ones attended to should be at least those which have the small cavities, thus keeping them from getting bad. A common expression of the writer's to such patients is as follows: "If for any reason you wish only two or three teeth filled, and those which need it the most, the writer would immediately, after careful inspection, fill the three teeth which had the smallest observable points of decay, thus preserving those teeth from becoming bad, and the very bad ones would only get a little worse." This will illustrate the position of the writer perhaps as well as could be otherwise expressed.

His observation has led him to believe that many of our specialty do not recognize the importance of thus acting. In fact, the writer's attention has been called, by several men of prominence, to the fact that by so doing one gets the name of being a high-priced dentist, and is thus marked for being shunned, when, as a matter of fact, such practice, while it causes much more work to be done at the time, saves a great amount of work in subsequent sittings of the same patient, as observed in a practice extending over a large number of years.

The writer believes that we have large responsibilities also in careful observation of the relations which are borne by the teeth, their eruption, care or lack of care upon the general health of the patient, as well as reflex disturbances which may be brought about by this irritation being reflected and observed in other of the special organs of the head. Cases could be cited which would illustrate this, and the writer thinks that this matter is generally accepted as true by the profession at large, but that sufficient care and thoroughness is not observed in a general way as to the minute observations of this rule in general practice.

Another responsibility which we face is that of supplying a masticating apparatus when the natural teeth and roots, or at least many of them, have been lost. Crown- and bridge-work may be made most useful as a conserver or restorer of health when a lack of mastication has caused disordered digestion and the accompanying ills, and yet a note of warning should be sounded that bridge-work or crowns may, by improper adjustment, be made to cause the very ills which they were intended to rectify. Very great care and thoughtfulness must be bestowed upon bridge-work and crowns to see that the articulation is made natural, and that the force of mastication may be upon the lines of natural resistance which would have obtained had the natural teeth not been lost. The writer has seen many bridges which were absolutely useless in themselves, and which have caused the loss of many of the abutments thereto, thus making the last condition of the patient worse than the first. Our effort should always be to advise and recommend those things which we feel assured will be for the permanent welfare of the patient, never allowing ourselves to be misled by any suggestion of his, or any wish to have cheap or over-expensive work done, if in our belief such work is unwarranted for permanent results.

